# Even lobar deposition of poorly soluble gold nanoparticles (AuNPs) is similar to that of soluble silver nanoparticles (AgNPs)

**DOI:** 10.1186/s12989-020-00384-w

**Published:** 2020-10-20

**Authors:** Hoi Pin Kim, Jin Kwon Kim, Mi Seong Jo, Jung Duck Park, Kangho Ahn, Mary Gulumian, Günter Oberdörster, Il Je Yu

**Affiliations:** 1 Aerosol Toxicology Research Center, HCTm CO.,LTD, Icheon, South Korea; 2 Department of Mechanical Engineering, Hanyang University, Ansan, South Korea; 3 Deparment of Preventive Medicine, College of Medicine, Chung-Ang University, Seoul, South Korea; 4 National Institute for Occupational Health, Johannesburg, South Africa; 5 Haematology and Molecular Medicine, University of the Witwatersrand, Johannesburg, South Africa; 6 Water Research Group, Unit for Environmental Sciences and Management, North West University, Private Bag X6001, Potchefstroom, 2520 South Africa; 7 Department of Environmental Medicine, University of Rochester, Rochester, NY USA; 8 HCT CO.,LTD, Seoicheon-ro 578 beon-gil, Majang-myeon, Icheon, 17383 South Korea

**Keywords:** Gold nanoparticles, Lung lobar deposition, Lung retention, Poorly soluble particles, Silver nanoparticles

## Abstract

**Background:**

Information on particle deposition, retention, and clearance is important when evaluating the risk of inhaled nanomaterials to human health. The revised Organization Economic Cooperation and Development (OECD) inhalation toxicity test guidelines now require lung burden measurements of nanomaterials after rodent subacute and sub-chronic inhalation exposure (OECD 412, OECD 413) to inform on lung clearance behavior and translocation after exposure and during post-exposure observation (PEO). Lung burden measurements are particularly relevant when the testing chemical is a solid poorly soluble nanomaterial. Previously, the current authors showed that total retained lung burden of inhaled soluble silver nanoparticles (AgNPs) could be effectively measured using any individual lung lobe.

**Methods and results:**

Accordingly, the current study investigated the evenness of deposition/retention of poorly soluble gold nanoparticles (AuNPs) after 1 and 5 days of inhalation exposure. Rats were exposed nose-only for 1 or 5 days (6 h/day) to an aerosol of 11 nm well-dispersed AuNPs. Thereafter, the five lung lobes were separated and the gold concentrations measured using an inductively coupled plasma-mass spectrophotometer (ICP-MS). The results showed no statistically significant difference in the AuNP deposition/retention among the different lung lobes in terms of the gold mass per gram of lung tissue.

**Conclusions:**

Thus, it would seem that any rat lung lobe can be used for the lung burden analysis after short or long-term NP inhalation, while the other lobes can be used for collecting and analyzing the bronchoalveolar lavage fluid (BALF) and for the histopathological analysis. Therefore, combining the lung burden measurement, histopathological tissue preparation, and BALF assay from one rat can minimize the number of animals used and maximize the number of endpoints measured.

## Background

OECD inhalation test guidelines have recently been revised to cover testing of nanoparticles, including toxicokinetic studies and lung burden measurements. For inhalation exposure, the first step of a toxicokinetic study is explained in revised inhalation toxicity guidelines 412 (subacute) and 413 (subchronic) [1, 2]. When the inhaled test nanomaterials are poorly soluble, OECD guidelines mandate that the lung burden should be measured to inform about the pulmonary-retained dose. Such lung burden measurements should be performed for all test chemicals within 24 h after exposure termination and at two or more additional post-exposure observation (PEO) intervals [1, 2]. However, the inclusion of two additional lung burden analyses for two additional post-exposure observation periods means an increased use of animals, from 40 to 120 animals for OECD 412 [1] and from 80 to 160 animals for OECD 413 [2]. In a previous report, we evaluated the variability of nanoparticle deposition in specific lung lobes of rats following inhalation exposure to soluble silver nanoparticles (AgNP, 20 nm). The results showed that 20 nm inhaled well-dispersed AgNPs were evenly deposited and retained in all lung lobes in terms of the Ag mass per gram of lung tissue [3]. Therefore, this suggests that any lung lobe can be used to determine the total lung burden, as long as the same lobe is sampled for these measurements. The remaining lung lobes can then be used for bronchoalveolar lavage fluid (BALF) collection and histopathological analysis, provided proper occlusion of the lung lobe is performed for the lung burden measurement. However, the results also indicated the need for further studies to determine the evenness of lobar retention in the case of poorly soluble nanoparticles. Accordingly, this report analyzes the particle deposition and retention in each lung lobe of a rat model to identify a representative lobe for lung burden measurements. Gold nanoparticles (AuNPs) with an average diameter of 11 nm were generated, as approximately 20–25% of inhaled particles of this size range are known to be deposited efficiently in the alveolar region with the Multiple-Path Particle Dosimetry Model (MPPD) and International Commission on Radiological Protection (ICRP) lung deposition models [4–6]. After 1 and 5 days of AuNP inhalation exposure, the rats were sacrificed and the lung lobes separated for lung burden analysis. The amounts retained in the lung lobes were then evaluated in terms of mass and particle number.

## Materials and methods

### Gold nanoparticle generation

The AuNPs used in this study were generated based on a well-established evaporation/condensation generation method, as previously described [3, 7–9] The generator consisted of a small ceramic heater connected to an AC power (112–113 V) supply and was housed within a quartz tube case (70 mm in diameter and 140 mm long). The heater dimensions were 50 × 5 × 1.5 mm, and a surface temperature of about 1500 °C within a local heating area of 5 × 10 mm^2^ was achieved within about 10s. Approximately 80 mg of the source material gold wire (purity 99.99%; 0.5 mm diameter, density 19.35 g/cm^3^, Higgslab Co., Ltd., South Korea) was positioned at the highest temperature point. The rats were exposed to the AuNP aerosol in a nose-only exposure chamber (30 ports, flow past, HCT, Icheon, Korea); 20 ports for animal exposure and 5 ports for chamber monitoring, including particle monitoring using a real-time particle monitor, sampling, and chamber environmental monitoring. Clean (dry and filtered) air was used as the carrier gas, and the gas flow was maintained at 25.0 L/min (Re = 572, laminar flow regime) using a mass flow controller (MFC, AERA, FC-7810CD-4 V, Japan) [10]. In the current study, the exposure system consisted of two chambers: AuNP exposure and fresh air control. The generator used 3 ± 0.1 LPM (liters per minute) for generating the aerosol and 22 ± 1 LPM for the aerosol dilution system. The total air flow volume in each chamber was 25 LPM and controlled by a mass flow controller (MFC, AERA, FC-7810CD-4 V, Japan). The flow rate to each port in the nose-only chamber was approximately 1 LPM, similar to the 0.75 L/min flow recommended by Pauluhn [11].

### Monitoring of inhalation chamber and analysis of AuNPs

The nanoparticle size distribution was measured in each chamber directly in real-time using a differential mobility analyzing system (DMAS); combining a differential mobility analyzer (DMA-20, 4220, range 6–225 nm, HCT Co., Ltd. Korea) and condensation particle counter (CPC, 3775, size range 4 nm- 3 μm, TSI INC., Shoreview, MN). Nanoparticles from 6 to 225 nm were measured using sheath air at 15 L/min and polydispersed aerosol air at 1.5 L/min for the DMAS and CPC, respectively. In addition, the mass concentration of AuNPs was determined chemically using an atomic absorption spectrophotometer (AAS, Perkin-Elmer 900 T, Waltham, MA, USA) after sampling on a mixed cellulose ester (MCE) filter (size: 37 mm and pore size 0.45 μm, SKC, UK) at a flow rate of 1.0 L/min [11]. Three samples were taken from the ports of the inhalation chamber each day during the 5-day exposure. The target mass concentration of generated AuNPs in the chambers was 40 μg/m^3^.

### Transmission Electron microscopy (TEM)

The TEM samples were collected on a TEM grid (200 mesh, Veco, Eerbeek, Holland) for 3 min using a nanocollector (HCT Co., Ltd., Icheon, Korea) in each chamber during the exposure period, and visualized under TEM (JEM-2100F, JEOL, Japan). The diameters of 200 randomly selected particles were measured at a magnification of 100,000, and the AuNPs analyzed using an energy-dispersive x-ray analyzer (EDX, Oxford. UK) at an accelerating voltage of 75 kV.

### Animals and exposure

Six-week-old male (20 rats; 213.64 ± 1.31 of weight (g); mean ± S.E), specific-pathogen-free (SPF) Sprague-Dawley rats were purchased from Orient Bio (Seong-nam, Korea) and acclimated for 2 weeks before starting the experiments. During the acclimation and experiment periods, the rats were housed in polycarbonate cages (5 rats per cage) in a room with controlled temperature (21.1 ± 0.33 °C) and humidity (48.8 ± 4.1%) with a 12-h light/dark cycle. The rats were fed a rodent diet (Woojung E&C, Seoul, Korea) and filtered water ad libitum. The rats were adapted to the nose-only tubes for a week with daily tube placement for 2 h. The 8-week-old rats, weighing 331.2 ± 2.8 g (20 rats mean ± SE), were then divided into 4 groups: fresh air control for 1 day (5 rats, 331.4 ± 5.5 g) and 5 days (5 rats; 331.8 ± 6.3 g), and AuNP exposed (6 h/day) for 1 day (5 rats, 331.3 ± 7.1 g) and 5 days (5 rats, 330.2 ± 5.4 g). The exposure concentration selected was a concentration that maximally generated from the generator and also based on our previous AuNP (13 nm) 5-day repeated inhalation exposure and AuNP (5 nm) 90-day repeated inhalation exposure studies [8, 12]. In the 5-day AuNP (13 nm) inhalation study at 13 μg/m^3^ did not show any noticeable inflammation and 90-day AuNP (5 nm) inhalation study at 0.04 to 20 μg/m^3^ showed minimal alveoli, an inflammatory infiltrate with a mixed cell type, and increased macrophages in the high-dose rats. Thus, our study concentration 35 μg/m^3^ for short-term exposure may not induce significant lung inflammation and result in sufficient tissue Au concentration by ICP-MS analysis. Therefore, histopathological evaluation and bronchoalveolar lavage fluid analysis that may exert particle deposition to the lung lobes were not conducted. The animals were examined daily on weekdays for any evidence of exposure-related effects, including respiratory, dermal, behavioral, nasal, or genitourinary changes suggestive of irritation. The body weights were evaluated at the time of purchase, at the time of grouping, once a week during the inhalation exposure, and before necropsy (results are not shown). The rat experiment was approved by the Hanyang University Institutional Animal Care and Use Committee in South Korea (HY-IACUC-2019-0049).

### Lung burden measurement

**Fig. 1:**
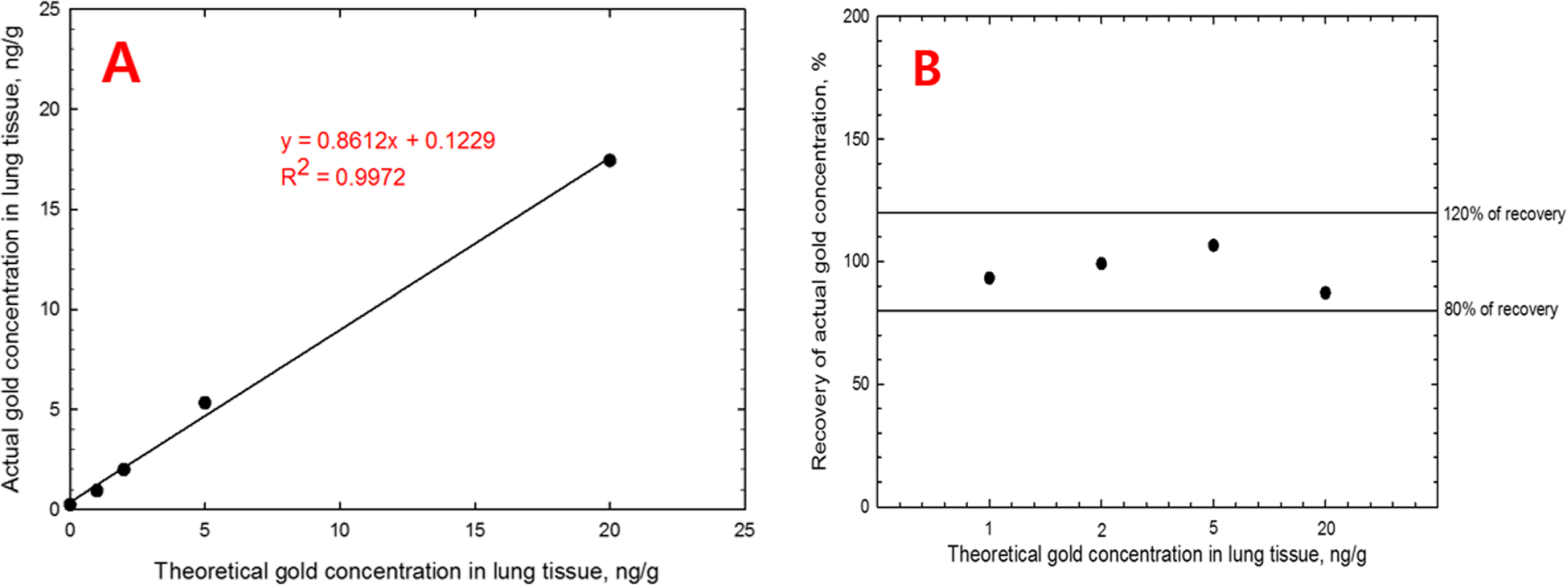
Results of spiked standard curve and recovery for AuNPs; **a**, spiked standard curve for gold nanoparticles (ng/g) in lung tissue. Analysis was y = 0.8612x + 0.1229 and R^2^ = 0.9972; **b**, Recovery results for gold nanoparticles in lung tissue. Recovery analysis for 1, 2, 5, and 20 ng/g was 93.24, 99.12, 106.54, and 87.24%, respectively

After 1 (6-h) or 5 days of exposure, the animals were immediately sacrificed by anesthetizing via an intraperitoneal injection of pentobarbital (EntobarVR, Hanlim Pharm Co. Ltd., Seoul, Korea) at a dose of 150 mg/kg body weight. The animals in the control group were sacrificed first and all the dissection instruments thoroughly washed with 70% ethyl alcohol between dissections. After incising the abdominal aorta, the thorax was opened by cutting up through the diaphragm and ribs. Once the lungs were isolated, the lung lobes were carefully separated and each lobe weighed and stored at -25 °C in a 25 mL conical tube. The wet weight of each lung lobe is presented in Table 2 (exposure for 1 day) and Table 3 (exposure for 5 days). The lung burden of AuNPs was determined from the lung content of gold analyzed using ICP-MS (PerkinElmer NEXION 300S, Concord, ON, Canada) based on the National Institute for Occupational Safety and Health (NIOSH) 7300 method [13]. The lung lobes were digested with 2 ml of concentrated nitric acid (purity of 69.0%; CAS.no of 7697-37-2, Fluka, Germany) using a microwave digestion system (MARS 230/60, CEM, Matthews, NC). Thereafter, the concentrations of gold in the digested solutions were analyzed using ICP-MS and calculated using a calibration curve prepared with standard gold solutions (0, 0.05, 0,2, 0,5 and 2 ppb). The spiked standard curve (0, 1, 2, 5, and 20 ng/g) and gold recovered from tissue were analyzed using the gold nanoparticles collected from the inhalation chamber and fresh lung tissue (non-exposed). The spiked standard curve for 0, 1, 2, 5, and 20 ng/g was analyzed as 0.24, 0.93, 1.98, 5.33, and 17.45 ng/g, respectively. The standard curve and R^2^ were y = 0.8612x + 0.1229 and 0.9972 of R^2^, respectively. The recoveries were 93.24% for 1 ng/g, 99.12% for 2 ng/g, 106.54% for 5 ng/g, and 87.24% for 20 ng/g. The limit of detection (LOD) and the limit of quantification (LOQ) were 13.61 ng/g and 41.23 ng/g, respectively. When analyzing the ICP-MS, the control samples included 10 dilutions using 0.1 HNO_3_, while the exposure samples included 1000 dilutions. The background gold for the digestion procedure was also validated using the same procedure with nitric acid only, where the background intensity was similar to the blank solution, 0.1% nitric acid. The recovery yields of AuNPs were 87.2–106.5%, as shown in Fig. 1. The digestion recovery of AuNPs from the lung tissue was calculated using Eq. 1

1 $$ \mathrm{Recovery}\ \left(\%\right)= measured\ concentration\ \left(\mathrm{ng}/\mathrm{g}\right)/\mathrm{spiked}\ \mathrm{concentration}\ \left(\mathrm{ng}/\mathrm{g}\right)\kern0.50em \mathrm{x}\ 100 $$

### Statistical analysis

An analysis of variance (ANOVA) test and Dunnett T3 multiple comparison tests were used to compare the gold content among the different lobes. The level of significance was set at *P* < 0.05.

## Results

### AuNP distribution and mass concentration

The AuNP aerosol total number concentration, CMD (count median diameter), GSD (geometric standard deviation), and surface area measured and calculated by the DMAS are all presented in Table 1.

**Table 1:** Distribution of AuNPs in inhalation chamber (mean ± SD)

AuNPs	Mean ± SD
1-day exposure number (#/cm^3^) ^a^	1.671 × 10^6^ ± 9.094 × 10^4^
5-day exposure number (#/cm^3^) ^a^	1.823 × 10^6^ ± 8.805 × 10^4^
CMD (nm), GSD ^a^	10.871, 1.426
Surface area (nm^2^/cm^3^) ^a^	8.972 × 10^8^ ± 4.742 × 10^7^
Volume (nm^3^/cm^3^) ^a^	2.445 × 10^9^ ± 1.725 × 10^8^
One particle mass (ng) ^a^	2.590 × 10^− 8^ ± 1.272 × 10^− 9^
Mass concentration (ug/m^3^) ^a^	47.212 ± 3.331
CMD^b^ (nm) and GSD	11.21, 1.43
AuNP mass concentration by AAS (μg/m^3^) ^C^	1-day exposure, 34.827 ± 3.635 5-day exposure, 34.679 ± 2.587

**a** Particle concentration and distribution were measured by DMAS; **b** CMD was measured by FE-TEM counting 200 particles and GSD was calculated by size of 84% / 50%; **c** AuNP mass concentration was analyzed by AAS; CMD, count median diameter; GSD, Geometric standard deviation; AAS, atomic absorption spectrometer; SMPS continually measured during 5-day exposure; 3 MCE filter samples per day were collected during exposure period and measured to determine mass concentration

**Fig. 2:**
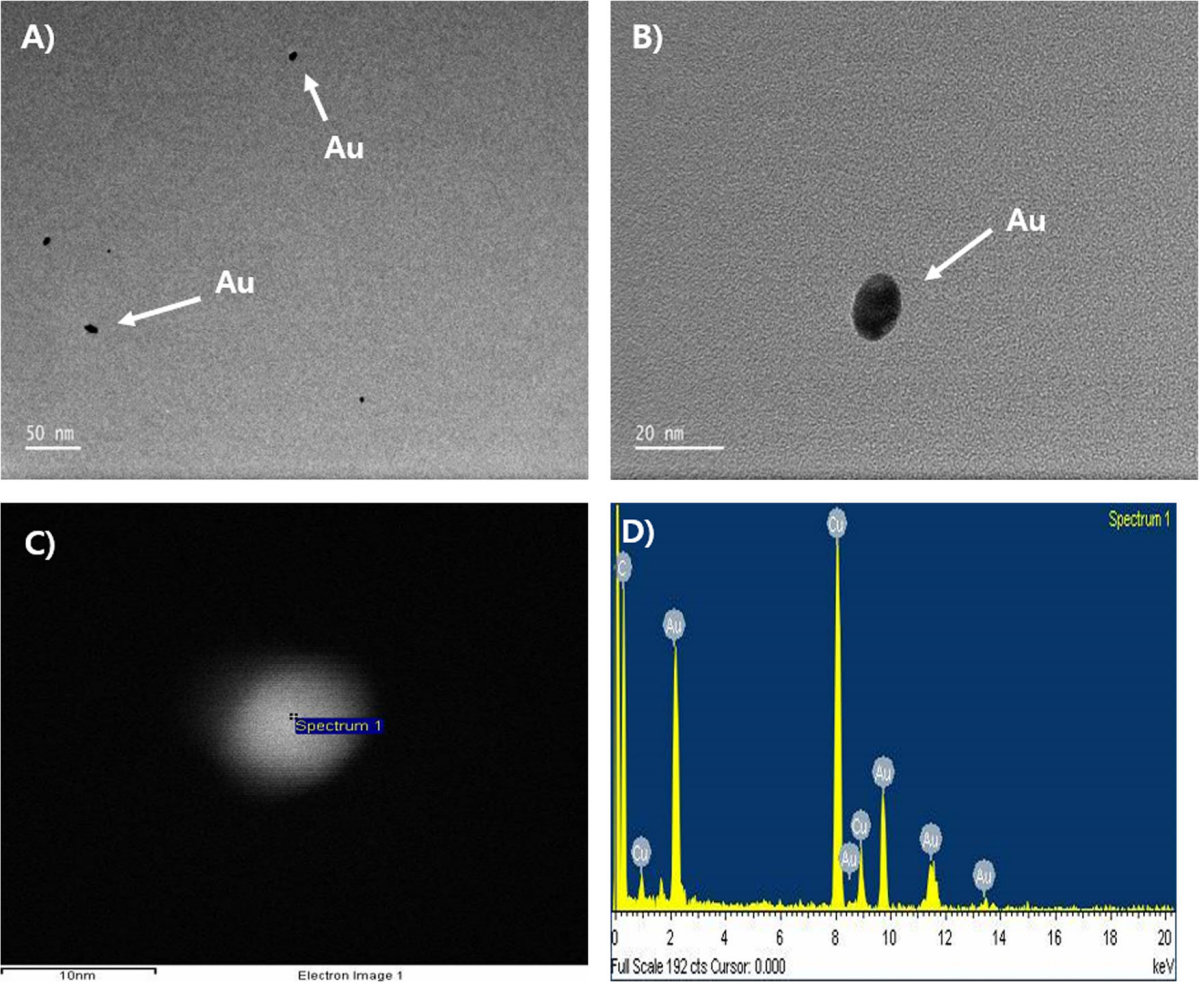
TEM of AuNPs sampled from nose-only chamber. **a**, Non-aggregated/agglomerated AuNPs (scale 50 nm); **b**, AuNP (scale20 nm); **c**, EDX analysis of particle (scale 50 nm), marked as ‘spectrum 1’; **d**, EDX results indicate approximately 100% gold

**Fig. 3:**
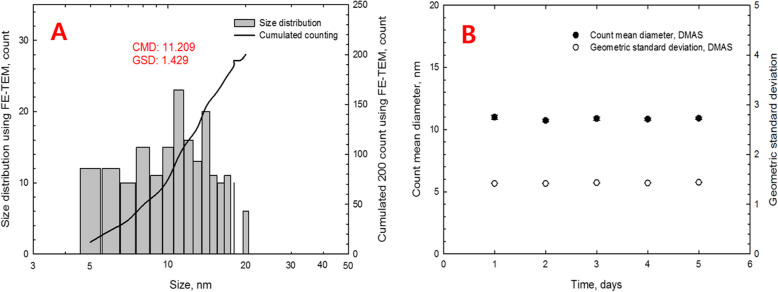
Size distribution of AuNPs by TEM counting and DMAS. Count median diameter (CMD), geometric standard deviation (GSD). **b**, particle CMD and GSD were measured by DMAS during 6 h/day over 5-day exposure period

**Fig. 4:**
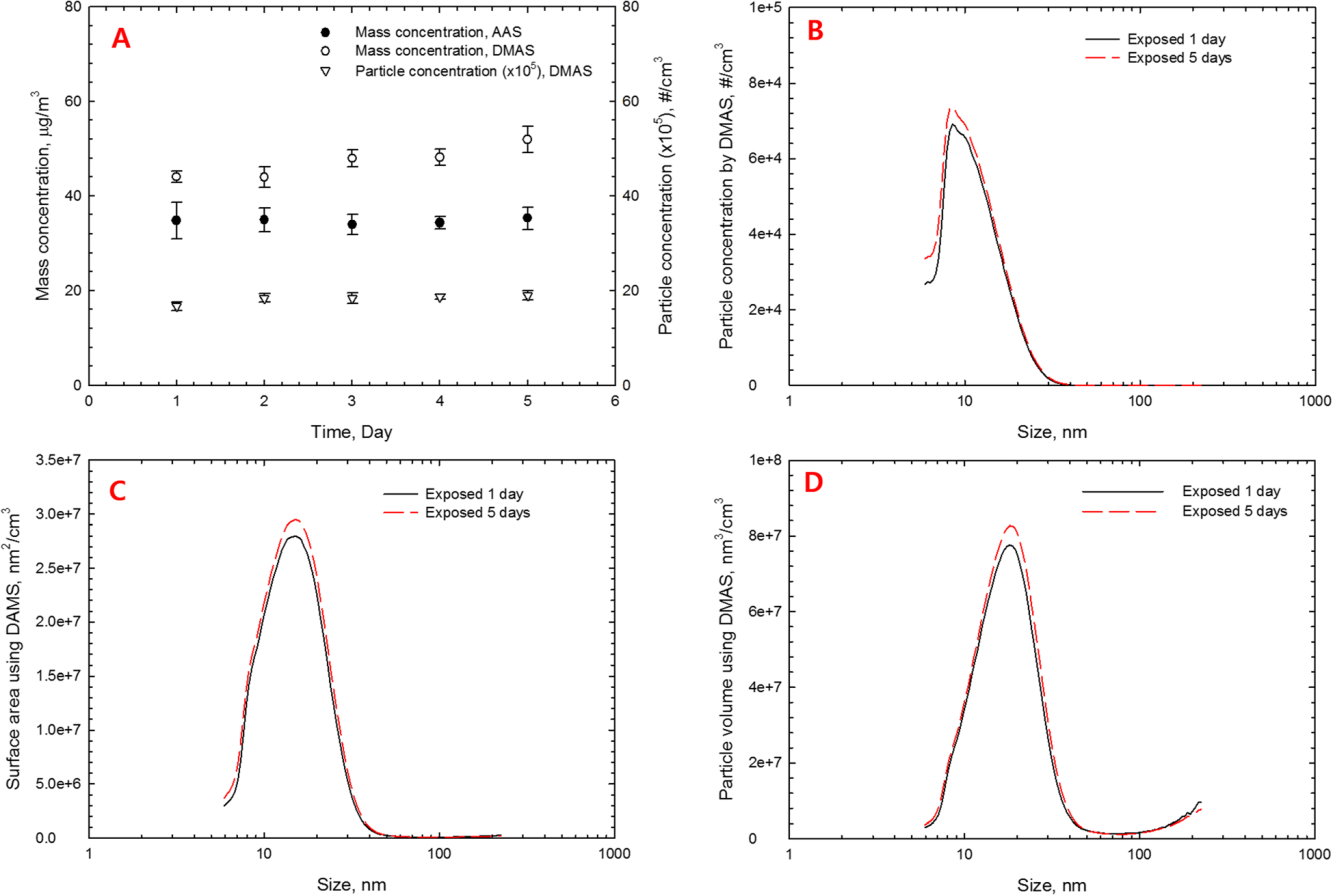
Particle diameter distribution of generated AuNPs in exposure chamber during 5-day exposure period by DMAS. **a**, particle diameter and GSD during exposure period; **b**, particle diameter distribution; **c**, particle surface area distribution; **d**, particle volume distribution. Samples were taken during 6 h/day over 5-day exposure period

The mass concentration estimated by the DMAS was 47.21 ± 3.33 μg/m^3^, while the mass concentration analyzed by the AAS after filter sampling was 34.7 ± 2.8 μg/m^3^ (Table 1). The AuNPs observed by TEM were spherical in shape and non-aggregated/agglomerated with CMD 11.21 nm and CMD 1.43 (Figs. 2 & 3, Table 1). The CMD and GSD were 10.87 and 1.59, respectively, and Fig. 4 shows the particle diameter, surface area, and volume distribution of the generated AuNPs in the exposure chamber during the 5-day exposure period.

### AuNP deposition/retention in lung lobes after 1-day (6-h) exposure

The AuNP deposition/retention per lobe is shown in Table 2, where the RCr (right cranial lobe), RM (right middle lobe), RCa (right caudal lobe), RA (right accessory lobe), and LL (left lung) retained 9.2, 14.4, 28.5, 12.0, 13.5, and 36.1% of the total lung deposition/retention, respectively; however, taken into account the different weights of the lung lobes, the AuNPs were evenly deposited/retained in terms of μg/g of lung tissue. (Table 2). The gold nanoparticle retention in the lung lobes after the 5-day exposure showed a similar retention pattern to that after the 1-day (6-h) exposure, with no statistically significant difference among the lobar concentration per unit weight. The retention percentages per lobe were also very similar after the 1-day and 5-day exposure periods. Finally, when comparing the deposition/retention for the 1-day exposure with the retention for the 5-day exposure, no notable clearance of AuNPs was observed in terms of the total lung deposition for the 5-day exposure period.

**Table 2:** Gold concentration in each lung lobe after 1-day exposure

Lobes	Retention (μg/g)^a^	Lung weight (g)^b^	Retention (μg)/lobe^c^
RCr	2.61 ± 0.62	0.11 ± 0.02	0.30 ± 0.08 (9.15%)
RM	3.36 ± 0.85	0.14 ± 0.02	0.47 ± 0.16 (14.42%)
RCa	2.85 ± 0.70	0.32 ± 0.02	0.92 ± 0.23 (28.42%)
RA	3.21 ± 0.59	0.12 ± 0.01	0.39 ± 0.10 (11.99%)
RL	2.98 ± 0.65	0.69 ± 0.05	2.07 ± 0.51 (63.98%)
LL	2.86 ± 0.68	0.41 ± 0.04	1.17 ± 0.29 (36.02%)
Total lung	2.94 ± 0.64	1.10 ± 0.09	3.24 ± 0.78

(mean ± S.D); 5 animals; No significant change for all contents; RCr, right cranial; RM, right median; RCa, right caudal; RA, right accessory lobe; RL, whole right lung (RCr + RM + RCa + RA); LL, left lung; Total lung, (RL + LL); a) μg/L (ICP-MS; ppb) × 0.005 L (end of volume) / lobe weight = gold concentration in 1 g; b) Each lobe weight; c) Retained mass concentration x lung lobe weight = gold concentration in lobe;

### AuNP deposition/retention in lung lobes after 5-day exposure

The retention trend for the 5-day exposure was very similar to that for the 1-day (6-h) exposure (Table 3), with no statistically significant difference in AuNP retention among the lobes. Plus, the retention percentages per lobe after the 5-day exposure were very similar to those after the 1-day exposure with no statistically significant difference. No significant clearance of AuNPs from the lung lobes was observed during the 5-day exposure, as the retention remained similar to that after the 1-day exposure (Table 3). Our previous study on clearance of AuNP after 5-day inhalation exposure showed that AuNPs were located in the alveolar region and macrophages throughout the 28-day recovery period (Supplement 1).

**Table 3:** Gold concentration in each lung lobe after 5-day exposure

Lobes	Retention (μg/g)^a^	Lung weight (g)^b^	Retention (μg)/lobe^c^
RCr	11.13 ± 2.44	0.11 ± 0.01	1.23 ± 0.21 (7.85%)
RM	15.64 ± 2.45	0.14 ± 0.01	2.17 ± 0.38 (13.86%)
RCa	14.85 ± 1.98	0.32 ± 0.02	4.70 ± 0.49 (30.06%)
RA	14.70 ± 3.93	0.13 ± 0.02	1.84 ± 0.46 (11.77%)
RL	14.39 ± 2.12	0.69 ± 0.04	9.93 ± 0.96 (63.54%)
LL	13.76 ± 2.63	0.42 ± 0.04	5.70 ± 0.55 (36.46%)
Total lung	14.12 ± 2.03	1.11 ± 0.08	15.63 ± 1.33

(mean ± S.D); 5 animals; * *P* < 0.05; comparing RCa, RL, LL, and total lung; RCr, right cranial; RM, right median; RCa, right caudal; RA, right accessory lobe; RL, whole right lung (RCr + RM + RCa + Ra); LL, left lung; Total lung, (RL + LL); a) μg/L (ICP-MS; ppb) × 0.005 L (end of volume) / lobe weight = gold concentration in 1 g; b) Each lobe weight; c) Retained mass concentration x lung lobe weight = gold concentration in lobe;

## Discussion

The newly revised OECD inhalation toxicity guidelines mandate bronchoalveolar lavage and lung burden measurements if the inhaled particles or nanoparticles are poorly soluble and likely to be retained in the lungs. This revision requires a 2–3-fold increase in the number of experimental animals used in order to collect sufficient information on the clearance kinetics, along with modifications to the inhalation toxicity equipment with more exposure ports for nose-only inhalation exposure and additional animal cages for whole-body exposure. The manual process involved in BAL fluid collection also means more labor-intensive experiments. As a result, all these additions incur extra financial costs. Therefore, in the case of nanomaterial inhalation, the current and previous papers by the present authors suggest halving the animal use and maximizing the endpoints by using one lobe of the right lung for the lung burden measurement. Demonstrating an even lobar deposition/retention of inhaled soluble silver nanoparticles in rat lungs implies extended application to poorly soluble or insoluble nanoparticles. Meanwhile, the mass concentration of AuNPs in each lung lobe also showed an even deposition, with no statistically significant difference in AuNP deposition after 6-h exposure or 5-day exposure in terms of gold mass/g of lung tissue. Another study also showed no statistically significant difference in soluble AgNP deposition among lobes after 6-h or 5-day exposure [3]. The only significant difference between AgNP and AuNP deposition/retention was AuNPs showed no significant clearance, whereas AgNPs showed approximately 30% clearance during the 5-day exposure period.

Several studies on the pulmonary deposition of 0.2–3.05 μm particles, including mono-dispersed (MMAD 1.5 μm) and poly-dispersed (MMAD 1.9 μm), found that the apical lobe of the right lung (RCr) received the highest deposition [14, 15]. Another particle deposition study using hamsters labeled with 99mTC (0.01–3.0 μm; 10% ≤0.1 μm; 70% 0.1–0.4 μm, and 4% ≥1.0 μm) resulted in a more even distribution with preferential deposition in the apical lobes [16]. When the particle deposition fractions in the nasal passages and in various lobes and regions of Long-Evans rat lungs were measured following nose-only exposure to 59Fe radiolabeled monodispersed condensation particles of triphenylphosphate, ranging from 0.9–4.2 μm, the left lung received a higher deposition than each lobe in the right lung. In the right lung, the caudal and accessory lobes showed the highest and lowest deposition, respectively, while the right medial and right cranial lobes showed similar depositions [17]. However, the main differences between the AgNP and AuNP studies by the current authors and other previous studies are the particle size and associated deposition mechanisms. The present study used a well-dispersed 11 nm AuNP aerosol that was deposited in the respiratory tract almost exclusively by diffusion, while other studies have used submicron to micron size particles with different deposition mechanisms, such as impaction and sedimentation. Therefore, the diffusion-dominant deposition mechanism of nanoparticles produced an even particle deposition among the lung lobes, representing a significant difference between nano-sized and micron-sized aerosols as regards even lung deposition.

Recent study by the current authors on the lung deposition and retention of multi-walled carbon nanotubes (MWCNTs) (MMAD 1.015 μm) after 28 days of inhalation and during 28 days post exposure showed that the lung clearance kinetics of MWCNTs could be effectively evaluated using one lobe from the right lung [18]. The BAL fluid was collected from the right lung after occluding the right caudal lobe (RCL) and left lung. The left lung was then used to evaluate the histopathology and the RCL to evaluate the lung burden [18]. As a result, the number of animals used was greatly reduced. Although the evenness of lobar deposition was not evaluated in that study, the results still suggested that the lung clearance kinetics could be effectively evaluated using a right lung lobe or consistently using the same lobe in the right lung. In another recent study, quantitative analyses of lung burdens on various shapes of carbon nanomaterials including carbon black (50 mg/m^3^), NM-401 (0.5 and 1.5 mg/m^3^), NM-403 (1.5 mg/m^3^) and MWCNT-7 (1.5 mg/m^3^) nanotubes were conducted after 28-day inhalation exposure. Their MMAD was 940 nm for printex-90 carbon black, 790 nm for NM-401, 1940 nm for NM-403, and 1780 nm for MWCNT. The median right lobe was separated and used for lung burden analysis successfully [19]. Therefore, the 3R principles (Reducing, Replacing, and Refining) may also be applied to fibrous MWCNTs, along with soluble AgNPs and insoluble AuNPs.

There is a concern whether the exposure duration used in this study, such as 5-day short-term exposure can simulate the standard of OECD inhalation toxicity subacute and subchronic tests and represent the subacute and subchronic exposure setting. Although there is no study on even lobar retention study after subacute and subchronic inhalation for nanomaterials, AuNP toxicokinetic study after acute exposure by Krelyling et al. [20] or short-term exposure (5-day) [12] or subacute exposure (28-day) conducted by our group (manuscript in revision) indicated that AuNP is insoluble, and has long elimination half-time, and eliminated very slowly with linear fashion. Elimination half-time of AuNP is (T_1/2_) 45 days [12] and our recent study AuNP 28-day exposure study indicated that elimination half-time is (T_1/2_) 81.5 days and elimination rate 0.0085/day (manuscript in submission). Therefore, the deposition and elimination of AuNP in 28-day exposure may be predictive with 5-day exposure. Therefore, similar deposition and lung burden pattern after subacute exposure would be expected as seen in a 5-day exposure study. Furthermore, silver nanoparticle (AgNP) subacute study conducted together with lung burden measurement according to the revised OECD test guideline 412 showed Ag clearance had two-phase clearance; fast and slow. The fast clearance, half-time ranging from 2 to 4 days, was the clearance for soluble Ag and the slow clearance, half-time ranging from 57 to 100 days, was the clearance of the  insoluble secondary AgNP generated from reacting the soluble Ag with biogenic molecules. The insoluble secondary AgNP had long half-time of elimination and eliminated very slowly with linear fashion [21]. Therefore, if similar deposition and clearance were expected for certain soluble and insoluble nanomaterials, lung burden or retention after short-term exposure could be extrapolated to subacute and subchronic exposure situations.

It should also be noted that the current findings of even nanoparticle deposition and retention in the lung lobes after inhalation were based on the useof ~ 11 nm AuNPs, ~ 20 nm AgNPs, and male Sprague-Dawley rats. Thus, caution is needed when using a single lung lobe to measure the lung burden and clearance following exposure to larger particles with impaction and sedimentation as the dominant deposition mechanisms. Moreover, longer-term inhalation studies with poorly soluble NPs or soluble NPs are needed to determine the evenness of lobar retention under such conditions, as the clearance may differ for different lung lobes. As such, the present findings may be limited to the particle size distribution, test material, and strain of animals used in this study. Despite these limitations, continuous efforts on reducing animals should be practiced.

## Conclusions

This study evaluated the evenness of deposition/retention of insoluble gold nanoparticles in rat lung lobes to confirm whether the results would follow the previously reported evenness of deposition/retention of soluble silver nanoparticles. The current results showed that 11 nm inhaled well-dispersed AuNPs were evenly deposited and retained in all lung lobes in terms of the Au mass per gram of lung tissue after 1 day and 5 days. Thus, the consistently even deposition and short-term retention of inhaled AuNPs per unit weight of rat lung lobe agreed well with the previous study using soluble Ag nanoparticles, indicating that any lung lobe can be used to predict the total lung burden, as long as the same lobe is sampled for the same measurement. This allows the remaining lung lobes to be used for BALF and histopathological analyses, provided proper occlusion of the lung lobe is performed for the lung burden measurement. Consequently, combining the lung burden measurement, histopathological tissue preparation, and BALF assay from one rat can minimize the number of animals used and maximize the number of endpoints measured.

## Supplementary information

**Additional file 1 Supplement 1**. Hyperspectral microscopic images of gold nanoparticles (13 nm and 105 nm) after 5-day inhalation exposure and post-exposure observations (3 and 28 day). Adopted from Fig. 10 of Han et al. (2015). Fig. 10. Small **a** and large **b** gold nanoparticles in rat lung sections observed by hyperspectral microscopy. *Arrows* indicate gold nanoparticles accumulated in multiple areas of lungs, including alveolar region and alveolar macrophages. **a** 28 days after small gold nanoparticle exposure; **b** 28 days after large gold nanoparticle exposure.

## Data Availability

All data and materials are included in the manuscript, Tables and Figures.
