# Supplementary material for: Even lobar deposition of poorly soluble gold nanoparticles (AuNPs) is similar to that of soluble silver nanoparticles (AgNPs)
Source: Part Fibre Toxicol. 2020 Oct 20;17:54. doi: 10.1186/s12989-020-00384-w (PMC7574491; doi:10.1186/s12989-020-00384-w)
Supplement: Supplementary file 1 — Additional file 1 Supplement 1. Hyperspectral microscopic images of gold nanoparticles (13 nm and 105 nm) after 5-day inhalation exposure and post-exposure observations (3 and 28 day). Adopted from Fig. 10 of Han et al. (2015). Fig. 10. Small a and large b gold nanoparticles in rat lung sections observed by hyperspectral microscopy. Arrows indicate gold nanoparticles accumulated in multiple areas of lungs, including alveolar region and alveolar macrophages. a 28 days after small gold nanoparticle exposure; b 28 days after large gold nanoparticle exposure. [file 12989_2020_384_MOESM1_ESM.docx]

Supplement 1. Hyperspectral microscopic images of gold nanoparticles (13 nm and 105 nm) after 5-day inhalation exposure and post-exposure observations (3 and 28 day). Adopted from Figure 10 of Han et al (2015). Figure 10. Small **a** and large **b** gold nanoparticles in rat lung sections observed by hyperspectral microscopy. *Arrows* indicate gold nanoparticles

accumulated in multiple areas of lungs, including alveolar region and alveolar macrophages. **a** 28 days after small gold nanoparticle exposure; **b** 28 days after large gold nanoparticle exposure
